# The Varied and Multifaceted Professional Roles of Today’s Nurses

**DOI:** 10.1097/JNR.0000000000000438

**Published:** 2021-05-13

**Authors:** Pei-Shan TSAI

The nursing profession is evolving rapidly to meet the challenges and opportunities presented by the constant, technology-driven changes in healthcare delivery and the ever-changing landscape of healthcare needs. In this issue of *The Journal of Nursing Research*, four articles address issues that influence professional identity formation in the nursing profession. Their topics include the effects of education on professional quality of life and health among nurses, the effects of film-based nursing education in developing professional nursing identity among nursing students, the impact of organizational support on practice outcomes in nurse practitioners, and the use of a function-focused interdisciplinary communication framework in a nursing home setting.

Evidence-based nursing interventions are paramount to the successful development and adoption of evidence-based practices in nursing. Two articles in this issue provide evidence regarding the efficacy of nursing interventions on different groups of nursing clients, including the efficacy of machine-based hand massage in patients awaiting outpatient surgery and the efficacy of auricula acupressure in community-dwelling poor sleepers.

Self-management is an important strategy for reducing chronic-disease-related symptom distress and illness burden. Also in this issue, the results of a survey querying the perceived effectiveness of pain self-management strategies in people with migraine headache are reported.

While high-quality quantitative studies are needed for evidence-based nursing practice, the qualitative research approach is useful for exploring issues and phenomena that are poorly defined or understood. To this end, the components of empowerment in family caregivers of community-dwelling people with dementia were also explored in a qualitative research study included in this issue.

Copyright © 2021 The Authors. Published by Wolters Kluwer Health, Inc. All rights reserved. This is an open access article distributed under the Creative Commons Attribution License 4.0 (CCBY), which permits unrestricted use, distribution, and reproduction in any medium, provided the original work is properly cited.

Address correspondence to: Pei-Shan TSAI, PhD, RN, Professor, School of Nursing, College of Nursing, Taipei Medical University, No. 250, Wu Xing St., Taipei City 11031, Taiwan, ROC. Tel: +886-2-27361661 ext. 6321; E-mail: ptsai@tmu.edu.tw

Cite this article as:

Tsai, P.-S. (2021). The varied and multifaceted professional roles of today’s nurses. *The Journal of Nursing Research, 29*(3), Article e147. https://doi.org/10.1097/jnr.0000000000000438

